# Long non‐coding RNA THRIL predicts increased acute respiratory distress syndrome risk and positively correlates with disease severity, inflammation, and mortality in sepsis patients

**DOI:** 10.1002/jcla.22882

**Published:** 2019-07-01

**Authors:** Yan'e Wang, Xiaoxing Fu, Bo Yu, Fen Ai

**Affiliations:** ^1^ Department of Emergency, The Central Hospital of Wuhan, Tongji Medical College Huazhong University of Science and Technology Wuhan China

**Keywords:** disease severity, inflammation, lnc‐THRIL, sepsis, survival

## Abstract

**Background:**

This present study aimed to investigate the correlation of long non‐coding RNA THRIL (lnc‐THRIL) with acute respiratory distress syndrome (ARDS) risk, disease severity, inflammation, and mortality in sepsis patients.

**Methods:**

A total of 109 sepsis patients admitted to intensive care units were consecutively recruited, and their blood samples were collected. After admission, patients were supervised and screened daily to identify the occurrence of ARDS. Clinical characteristics, routine laboratory testing, and disease severity were recorded, and all enrolled patients were followed up until death in the hospital or discharge for mortality records. Lnc‐THRIL was detected by quantitative polymerase chain reaction, and inflammatory cytokine levels were measured by human enzyme‐linked immunoassay.

**Results:**

A total of 32 (29.4%) sepsis patients occurred ARDS and 77 (71.6%) did not. Lnc‐THRIL was upregulated in ARDS group compared with non‐ARDS group, and it had good value in distinguishing ARDS from non‐ARDS in sepsis patients (AUC: 0.706; 95%CI: 0.602‐0.809). Besides, lnc‐THRIL, smoke, and chronic obstructive pulmonary disease independently predicted increased risk of ARDS. As for disease severity, lnc‐THRIL positively correlated with APACHE II score and SOFA score in sepsis patients. Regarding inflammation, lnc‐THRIL was positively associated with CRP, PCT, TNF‐α, and IL‐1β levels in sepsis patients. Additionally, the mortality rate was 30.2%, and lnc‐THRIL was upregulated in non‐survivors compared with survivors, presenting a good value (AUC: 0.780; 95%CI: 0.683‐0.876) in predicting mortality in sepsis patients.

**Conclusion:**

Lnc‐THRIL predicts increased risk of ARDS and positively correlates with disease severity, inflammation, and mortality in sepsis patients.

## 1 INTRODUCTION

Sepsis is defined as life‐threatening organ dysfunction resulted from dysregulated host immune response and systemic inflammation to microbial infection.^1^ It is estimated to influence more than 19 million people each year globally, and the in‐hospital mortality rates are around 15%‐25% for sepsis patients.^2^, ^3^ Pulmonary dysfunction such as acute respiratory distress syndrome (ARDS) is one of the predominant organ dysfunctions caused by systemic inflammation, uncontrolled cytokine, or release of other mediators, which not only contributes to the high mortality of sepsis but also reduces the quality of life for sepsis survivors.^4^, ^5^ Although our understanding about the pathogenesis of sepsis has progressed with the focus shifting from pathogenicity of microorganisms to the host inflammation and immune response, it does not substantially lead to improvement of treatment outcomes of sepsis. Instead, the management of sepsis is still dependent on early recognition and adherence to the current standard treatment.^1^ Therefore, exploration of biomarkers for end‐organ dysfunction and novel therapeutic targets for sepsis is essential to improve the prognosis of sepsis patients.

Long non‐coding RNA (lncRNA) is a class of RNAs longer than 200 nucleotides and does not contain protein‐coding sequences.^6^ Accumulating studies reveal that a number of lncRNAs are implicated in regulating gene transcription as well as etiology of immune or inflammatory diseases.^7^, ^8^ Tumor necrosis factor (TNF)‐related and heterogeneous nuclear ribonucleoprotein l (hnRNPL)‐related immunoregulatory lncRNA (lnc‐THRIL) is responsible for the transcription of TNF‐α.^9^ Clinical studies show that lnc‐THRIL is involved in etiology of inflammatory diseases such as Kawasaki disease and systemic lupus erythematosus (SLE).^9^, ^10^ Moreover, lnc‐THRIL is also closely related with inflammation‐induced cell injury, and it is critical for the development of ARDS when the injury of alveolar cells is severe, leading to increased permeability pulmonary edema in the lung.^11^, ^12^ However, the function of lnc‐THRIL in sepsis (a typical immune response‐induced inflammatory disease) or its correlation with ARDS in sepsis patients is still not clear. Thus, we hypothesized that lnc‐THRIL might participate in the pathogenesis of sepsis and contribute to ARDS risk in sepsis patients as well, and we aimed to investigate the correlation of lnc‐THRIL with the risk of ARDS, disease severity, inflammation as well as mortality in sepsis patients.

## 2 MATERIALS AND METHODS

### 2.1 Patients

From July 2016 to June 2018, 109 sepsis patients admitted to intensive care units (ICUs) of The Central Hospital of Wuhan were consecutively recruited in this study. The inclusion criteria were as follows: (a) diagnosed as sepsis according to the Surviving Sepsis Campaign: International Guidelines for Management of Severe Sepsis and Septic Shock, 2012^13^; (b) age above 18 years old. The exclusion criteria were as follows: (a) infected with human immunodeficiency virus; (b) died within 24 hours after admitted in ICU; and (c) complicated with hematopoietic system disease or malignancies. Besides, pregnant or lactating women were excluded from the current study as well. After admission, patients were supervised and screened daily to identify those with sepsis‐associated acute respiratory distress syndrome (ARDS) according to the Berlin definition of ARDS,^14^ and there were 32 patients developing ARDS during hospitalization. Furthermore, all enrolled patients were followed up until death in the hospital or discharge, and a total of 33 patients died in the in the hospital, which were defined as non‐survivors, and 76 patients survived, which were defined as survivors. Written informed consents were obtained from all patients or their guardians after the study was approved by the Ethics Committee of the Hospital.

### 2.2 Samples collection

Blood samples of sepsis patients were collected within 24 hours after admission. The samples were divided into two aliquots, one was centrifuged at room temperature for separation of serum, and the other was centrifuged at 4°C for separation of plasma. Both serum and plasma were separated within 1 hour after blood samples collection and then stored at −80°C until determination.

### 2.3 Determination of lnc‐THRIL

Total RNA was extracted from plasma samples using TRIzol Reagent (Invitrogen, USA) and was reversely transcribed into cDNA using PrimerScript Real‐Time Reagent Kit (TaKaRa, Japan). qPCR was then performed using SYBR Premix Ex TaqTM II (TaKaRa, Japan) to quantify the expression of lnc‐THRIL, and GAPDH was used as internal reference. Primers were as follows: lnc‐THRIL, forward: AACTTCACAGGAACACTACACAAGA, reverse: TAGGCAACAGAGCAAGACTTCATC; GAPDH, forward: GAGTCCACTGGCGTCTTCAC, reverse: ATCTTGAGGCTGTTGTCATACTTCT.

### 2.4 Determination of inflammatory cytokines

Human enzyme‐linked immunoassay (ELISA) kits (Abcam, Massachusetts, USA) were applied to determine the level of inflammatory cytokines in serum, including tumor necrosis factor (TNF‐α), interleukin‐1β (lL‐1β), and IL‐17. And all procedures of ELISA were performed in accordance with the manufacturer's instructions.

### 2.5 Data collection

Patients’ clinical and routine laboratory testing data were collected after enrollment, which included (a) clinical characteristics: age, gender, body mass index (BMI), smoke, chronic comorbidities (such as chronic obstructive pulmonary disease (COPD), cardiomyopathy, chronic kidney failure, and cirrhosis); (b) routine laboratory testing: serum creatinine (Scr), albumin, white blood cell (WBC), C‐reactive protein (CRP), and procalcitonin (PCT). Moreover, acute physiology and chronic health evaluation (APACHE) II score used as an assessment of the severity of sepsis and the sequential organ failure assessment (SOFA) score used for the assessment of organ failure were also collected, which were evaluated within 24 hours after admission.

### 2.6 Statistical analysis

GraphPad Prism 7.00 (GraphPad Software, San Diego, USA) was used to make figures, and SPSS 22.0 (SPSS Inc, Chicago, USA) was used for statistical data processing. Count data were expressed as count or count (percentage); quantitative data were presented as mean ± SD if normally distributed or median (25th‐75th quantiles) if not normally distributed. The difference of lnc‐THRIL relative expression between two groups was determined by Wilcoxon rank sum test; correlation of lnc‐THRIL relative expression with disease severity as well as inflammatory cytokines was determined by Spearman correlation analysis. Receiver‐operating characteristic (ROC) curves and the area under the ROC curve (AUC) were used to assess the value of lnc‐THRIL relative expression in distinguishing ARDS from non‐ARDS and survivors from non‐survivors. Univariate and multivariate logistic regression model analyses were performed to assess factors predicting ARDS *P* value <0.05 was considered significant.

## 3 RESULTS

### 3.1 Patients’ characteristics

The mean age of 109 sepsis patients was 58.0 ± 10.7 years, and there were 80 males and 29 females. The numbers of patients who smoke, chronically complicated with COPD, cardiomyopathy, chronic kidney failure, and cirrhosis were 37(33.9%), 17 (15.6%), 38 (34.9%), 11 (10.1%), and 21 (19.3%), respectively. The mean APACHE II score and SOFA score were 16.3 ± 5.9 and 8.8 ± 4.2, respectively. Other detailed patients’ characteristics were listed in Table 1.

**Table 1:** Characteristics of sepsis patients

Characteristics	Sepsis patients (N = 109)
Age (y)	58.0 ± 10.7
Gender (male/female)	80/29
BMI (kg/m^2^)	23.4 ± 4.8
Smoke (n/%)	37 (33.9)
Chronic comorbidities (n/%)	
COPD	17 (15.6)
Cardiomyopathy	38 (34.9)
Chronic kidney failure	11 (10.1)
Cirrhosis	21 (19.3)
Scr (mg/dL)	1.50 (1.09‐2.07)
Albumin (g/L)	27.14 (21.65‐37.07)
WBC (10^9^/L)	13.32 (3.19‐29.78)
CRP (mg/L)	91.13 (50.53‐140.03)
PCT (ng/mL)	13.65 (8.40‐24.40)
APACHE II score	16.3 ± 5.9
SOFA score	8.8 ± 4.2
TNF‐α (pg/mL)	166.14 (107.05‐253.05)
IL‐1β (pg/mL)	10.22 (4.96‐21.76)
IL‐17 (pg/mL)	156.94 (74.27‐258.67)

Data were presented as mean value ± standard deviation, count (percentage), or median (25th‐75th quantiles). APACHE, acute physiology and chronic health evaluation; BMI, body mass index; COPD, chronic obstructive pulmonary disease; CRP, C‐reactive protein; IL, interleukin; PCT, procalcitonin; Scr, serum creatinine; SOFA, sequential organ failure assessment; TNF, tumor necrosis factor; WBC, white blood cell.

### 3.2 Upregulation of lnc‐THRIL in sepsis patients developed ARDS

**Figure 1:**
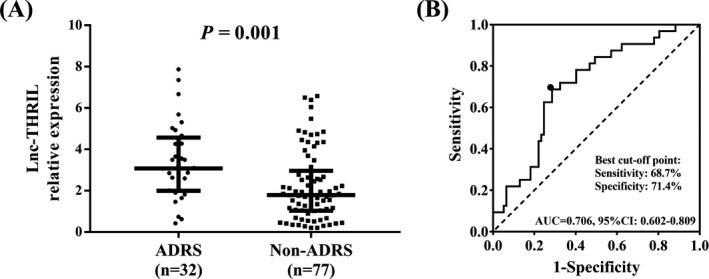
Lnc‐THRIL relative expression in ARDS and non‐ARDS. Lnc‐THRIL relative expression was higher in sepsis patients developed ARDS compared with those who did not (A) and had good value in distinguishing ARDS from non‐ARDS (B). Difference of lnc‐THRIL relative expression between two groups was determined by Wilcoxon rank sum test. ROC curve and the AUC were used to assess the value of lnc‐THRIL relative expression in distinguishing ARDS from non‐ARDS *P* < 0.05 was considered significant. ARDS, acute respiratory distress syndrome; AUC, area under ROC curve; ROC, receiver‐operating characteristic

The occurrence rate of ARDS was 29.4% in sepsis patients during hospitalization. And patients were allocated to ARDS group (N = 32) or non‐ARDS group (N = 77) depending on whether their developed ARDS or not (Figure 1A). The expression of lnc‐THRIL was elevated in ARDS group (3.082 [1.997‐4.568]) compared with non‐ARDS group (1.791 [1.027‐2.962]). ROC curve illustrated that lnc‐THRIL was of good value in distinguishing ARDS from non‐ARDS in sepsis patients with AUC of 0.706 (95%CI: 0.602‐0.809; Figure 1B). The sensitivity and specificity were 68.7% and 71.4%, respectively, and the lnc‐THRIL relative expression was 2.583 at the best cut‐off point. The best cut‐off point was the point where the sum of sensitivity and specificity reached the maximum.

### 3.3 Factors predicting ARDS in sepsis patients

Univariate logistic regression model analysis exhibited that lnc‐THRIL relative expression (OR = 1.427, *P = *0.003), age (OR = 1.041, *P = *0.045), smoke (OR = 3.925, *P* = 0.002), COPD (OR = 4.545, *P = *0.006), CRP (OR = 1.006, *P* = 0.029), and APACHE II score (OR = 1.114, *P* = 0.007) were correlated with increased risk of ARDS in sepsis patients (Table 2). Multivariate logistic regression further disclosed that lnc‐THRIL relative expression (OR = 1.511, *P = *0.034), smoke (OR = 3.983, *P* = 0.017), and COPD (OR = 9.193, *P = *0.005) independently predicted increased risk of ARDS in sepsis patients.

**Table 2:** Univariate and multivariate logistic regression model analysis of factors predicting ARDS

Items	Univariate logistic regression				Multivariate logistic regression			
	*P* value	OR	95% CI		*P* value	OR	95% CI	
			Lower	Higher			Lower	Higher
Lnc‐THRIL relative expression	0.003	1.427	1.125	1.809	0.034	1.511	1.031	2.214
Age	0.045	1.041	1.001	1.084	0.165	1.042	0.983	1.103
Gender (male)	0.817	0.897	0.356	2.259	0.940	1.052	0.284	3.893
BMI	0.378	1.040	0.954	1.133	0.348	1.067	0.932	1.222
Smoke	0.002	3.925	1.645	9.365	0.017	3.983	1.281	12.383
COPD	0.006	4.545	1.546	13.360	0.005	9.193	1.932	43.750
Cardiomyopathy	0.945	0.970	0.408	2.308	0.650	1.340	0.379	4.740
Chronic kidney failure	0.592	1.429	0.388	5.264	0.360	0.378	0.047	3.028
Cirrhosis	0.536	0.706	0.235	2.125	0.070	0.224	0.044	1.132
Scr	0.192	1.205	0.911	1.594	0.588	1.146	0.700	1.878
Albumin	0.418	0.982	0.939	1.026	0.479	0.968	0.886	1.059
WBC	0.215	1.017	0.990	1.044	0.975	0.999	0.949	1.052
CRP	0.029	1.006	1.001	1.012	0.918	1.000	0.992	1.008
PCT	0.101	1.023	0.996	1.050	0.326	1.019	0.981	1.058
APACHE II score	0.007	1.114	1.031	1.204	0.235	1.107	0.936	1.309
SOFA score	0.127	1.079	0.979	1.188	0.252	0.892	0.734	1.084
TNF‐α	0.265	1.002	0.999	1.005	0.876	1.000	0.995	1.005
IL‐1β	0.077	1.026	0.997	1.057	0.294	1.021	0.982	1.063
IL‐17	0.267	1.001	0.999	1.003	0.674	1.001	0.997	1.004

Factors predicting ARDS were determined by univariate and multivariate logistic regression analyses. *P* value < 0.05 was considered significant. ARDS, acute respiratory distress syndrome; BMI, body mass index; COPD, chronic obstructive pulmonary disease; APACHE, acute physiology and chronic health evaluation; SOFA, sequential organ failure assessment; Scr, serum creatinine; WBC, white blood cell; CRP, C‐reactive protein; PCT, procalcitonin; TNF, tumor necrosis factor; IL, interleukin.

### 3.4 Correlation of lnc‐THRIL with disease severity in sepsis patients

**Figure 2:**
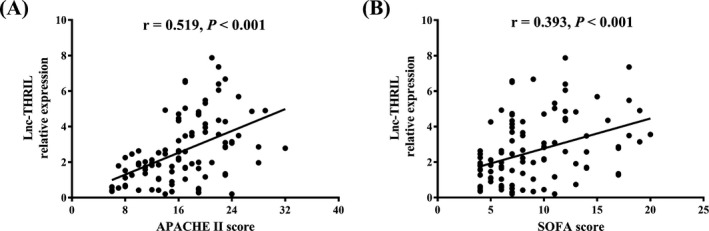
Correlation of Lnc‐THRIL relative expression with the severity of the patients. Lnc‐THRIL relative expression was positively correlated with APACHE II score (A) and SOFA score (B). Correlation of lnc‐THRIL relative expression with disease severity was determined by Spearman correlation analysis. *P* < 0.05 was considered significant. APACHE, acute physiology and chronic health evaluation; SOFA, sequential organ failure assessment

Spearman test illustrated that lnc‐THRIL relative expression was positively correlated with APACHE II score (*r* = 0.519, *P* < 0.001) (Figure 2A) and SOFA score (*r* = 0.393, *P* < 0.001) (Figure 2B) in sepsis patients.

### 3.5 Correlation of lnc‐THRIL with inflammatory markers in sepsis patients

**Figure 3:**
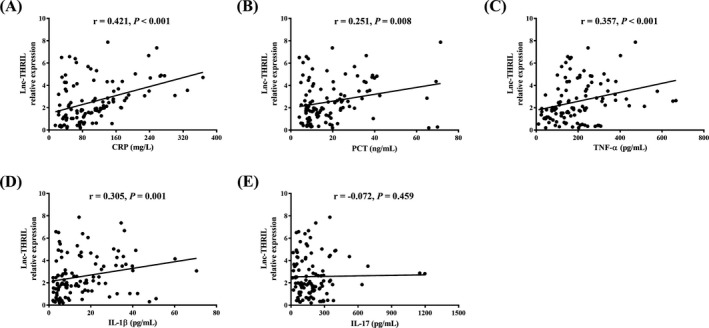
Correlation of Lnc‐THRIL relative expression with inflammation. Lnc‐THRIL relative expression was positively correlated with CRP (A), PCT (B), TNF‐α (C), and IL‐1β (D) but not with IL‐17 (E). Correlation of lnc‐THRIL relative expression with inflammatory cytokines was determined by Spearman correlation analysis. *P* < 0.05 was considered significant. CRP, C‐reactive protein; IL, interleukin; PCT, procalcitonin; TNF, tumor necrosis factor

Lnc‐THRIL relative expression was positively correlated with levels of CRP (*r* = 0.421, *P* < 0.001) (Figure 3A), PCT (*r* = 0.251, *P* = 0.008) (Figure 3B), TNF‐α (*r* = 0.357, *P* < 0.001) (Figure 3C), and IL‐1β (*r* = 0.305, *P* = 0.001) (Figure 3D), but not correlated with IL‐17 level (*r *= −0.072, *P* = 0.459) (Figure 3E) in sepsis patients.

### 3.6 Upregulation of lnc‐THRIL in non‐survival sepsis patients

**Figure 4:**
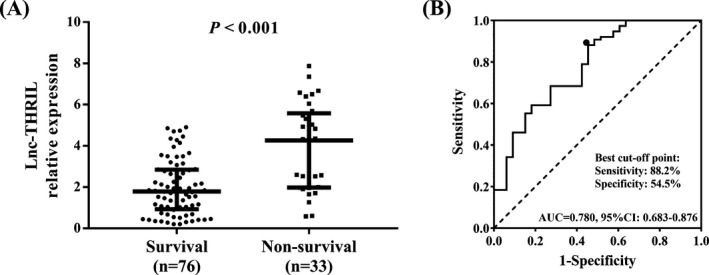
Lnc‐THRIL relative expression in survival and non‐survival. Lnc‐THRIL relative expression was inhibited in survivors compared with non‐survivors (A) and was of good value in distinguishing survivors from non‐survivors (B). Difference of lnc‐THRIL relative expression between two groups was determined by Wilcoxon rank sum test. ROC curves and the AUC were used to assess the value of lnc‐THRIL relative expression in distinguishing ARDS from non‐ARDS *P* < 0.05 was considered significant. AUC, area under ROC curve; ROC, receiver‐operating characteristic

There were 76 survivors and 33 non‐survivors in all sepsis patients, and the mortality rate was 30.2%. Lnc‐THRIL relative expression was elevated in non‐survivors (4.262 [1.983‐5.582]) compared with survivors (1.784 [0.928‐2.849]) (Figure 4A), and it presented a great value in predicting mortality in sepsis patients with AUC of 0.780 (95%CI: 0.683‐0.876) (Figure 4B). The sensitivity and specificity were 88.2% and 54.5%, respectively, at the best cut‐off point, and lnc‐THRIL relative expression was 3.671. The best cut‐off point was the point where the sum of sensitivity and specificity reached the maximum.

### 3.7 Subgroup analysis

**Figure 5:**
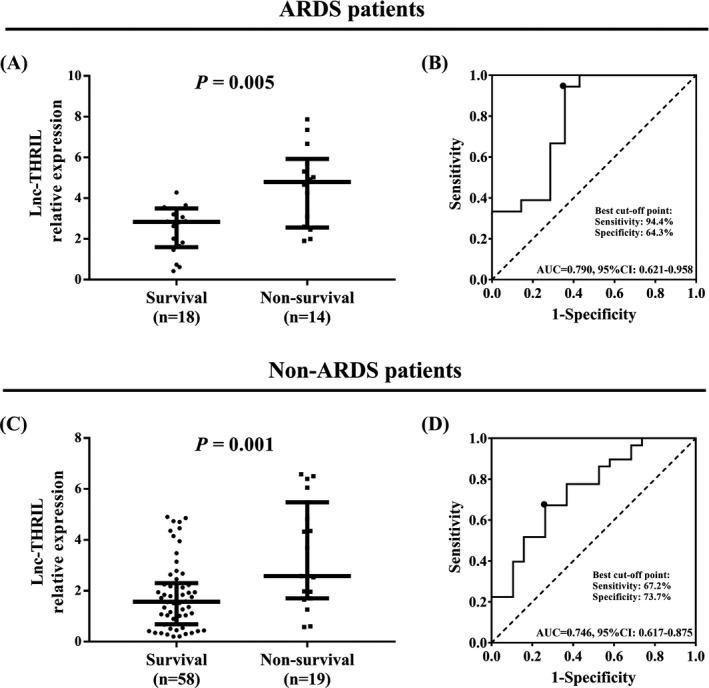
Subgroup analysis of lnc‐THRIL relative expression in survival and non‐survival. For patients who developed ARDS, lnc‐THRIL relative expression was reduced in survivors compared with non‐survivors (A) and had good value in telling survivors from non‐survivors (B). For patients did not develop ARDS, lnc‐THRIL relative expression was decreased in survivors compared with non‐survivors (C) and was of good value in distinguishing survivors from non‐survivors (D). Difference of lnc‐THRIL relative expression between two groups was determined by Wilcoxon rank sum test. ROC curves and the AUC were used to assess the value of lnc‐THRIL relative expression in distinguishing non‐survivors from survivors. *P* < 0.05 was considered significant. ARDS, acute respiratory distress syndrome; AUC, area under ROC curve; ROC, receiver‐operating characteristic

Additionally, sepsis patients were subdivided into ARDS group and non‐ARDS group, and correlations of lnc‐THRIL with disease severity in subgroups were determined (Table 3). In ARDS group, lnc‐THRIL was positively correlated with APACHE II score (*r* = 0.518, *P* = 0.002) and SOFA score (*r* = 0.364, *P* = 0.041). Also, in non‐ARDS group, positive association of lnc‐THRIL with APACHE II score (*r* = 0.411, *P* < 0.001) and SOFA score (*r* = 0.323, *P* = 0.004) was observed. Besides, the expression of lnc‐THRIL in survivors and non‐survivors, as well as its predictive value on mortality, was evaluated in subgroup analysis as well. In ARDS patients, lnc‐THRIL was downregulated in survivors (N = 18) (2.840 [1.600‐3.493]) compared with non‐survivors (N = 14) (4.800 [2.560‐5.931]) (Figure 5A), and it had a good value in telling survivors from non‐survivors (AUC = 0.790, 95%CI: 0.621‐0.958; Figure 5B). As for in non‐ARDS patients, lnc‐THRIL expression was lower in survivors (N = 58) (1.570 [0.683‐2.303]) compared with non‐survivors (N = 19) (2.573 [1.700‐5.480]) (Figure 5C), and it was of good value in distinguishing survivors from non‐survivors (AUC = 0.746, 95%CI: 0.617‐0.875; Figure 5D).

**Table 3:** Correlation of lnc‐THRIL relative expression with disease severity of sepsis patients

Items	Lnc‐THRIL relative expression	
	*P *value	Correlation coefficient (*r*)
ARDS patients		
APACHE II score	0.002	0.518
SOFA score	0.041	0.364
NON‐ARDS patients		
APACHE II score	<0.001	0.411
SOFA score	0.004	0.323

Correlation of lnc‐THRIL relative expression with disease severity of the patients was determined by Spearman rank correlation test. *P* value < 0.05 was considered significant. ADRS, acute respiratory distress syndrome; APACHE, acute physiology and chronic health evaluation; SOFA, sequential organ failure assessment.

## 4 DISCUSSION

From the present study, we observed that: (a) 29.4% of sepsis patients developed ARDS during hospitalization, and lnc‐THRIL independently predicted increased risk of ARDS; (b) lnc‐THRIL was positively correlated with disease severity and inflammatory cytokine levels; and (c) lnc‐THRIL was also upregulated in non‐survivors in sepsis patients.

LncRNAs are diverse and abundant in each component of human life with the total number exceeding that of protein‐coding genes.^7^ The biological complexity of lncRNAs enables them to participate in various biological processes such as gene transcription, cell state coordination, and disease progression, while only a small proportion of lncRNAs are studied.^6^ As one of the novel lncRNAs, lnc‐THRIL is initially disclosed to regulate the expression of TNF‐α, which is a key mediator of inflammation and immune response by forming a complex with hnRNPL and then binding to the promoter region of TNF‐α, and it influences the expression of inflammatory cytokines such as IL‐1β, IL‐6, and IL‐8.^9^, ^11^ Additionally, lnc‐THRIL is reported to influence inflammation‐related cell injuries in inflammatory diseases, and cell injury, especially alveolar epithelial cells injury that is one of the main contributors for the development of ARDS in sepsis.^11^, ^12^ Considering that sepsis is an immune‐induced inflammatory disease, and ARDS in sepsis is largely attributed to inflammation‐induced injury to the cells in the lung, we hypothesized that lnc‐THRIL might influence ARDS in sepsis. Our data revealed that 29.4% of sepsis patients developed ARDS during hospitalization, and lnc‐THRIL was upregulated in sepsis patients developed ARDS with good value in distinguishing ARDS from non‐ARDS in sepsis patients. The possible reason was that upregulated lnc‐THRIL might induce the release of TNF‐α as well as the subsequent boost of inflammatory mediators, which might elevate the level of radicals in lung microcirculation to injure the alveolar epithelial cells, thereby disrupted the barrier of lung microcirculation and subsequently increased the risk of ARDS in sepsis patients.^12^

There have been only a few studies revealing the aberrant expression and function of lnc‐THRIL in patients with inflammatory and auto‐immune diseases. For instance, lnc‐THRIL is upregulated in multiple sclerosis patients compared with healthy controls.^15^ And lnc‐THRIL is clearly lower in the acute phase compared with convalescent‐phase in patients with Kawasaki disease.^9^ The close association of lnc‐THRIL with Kawasaki disease and multiple sclerosis as well as its correlation with TNF‐α implicate that it may be involved in other inflammatory diseases, while it is not studied in sepsis yet. Therefore, we were interested in whether lnc‐THRIL was involved in the pathogenesis of sepsis or not, and discovered that lnc‐THRIL was positively correlated with APACHE II score and SOFA score in sepsis patients. This might be attributed to that lnc‐THRIL might initiate the transcription of TNF‐α and thereby promote the inflammatory response in sepsis patients, which aggravated disease condition in sepsis patients. The positive correlation of lnc‐THRIL with inflammation in sepsis patients was verified in our following analysis.

Although the function of lnc‐THRIL in regulating inflammation and immune responses has been disclosed, clinical studies investigating the correlation of lnc‐THRIL with inflammatory markers levels in patients with inflammatory diseases are still obscure. Considering that the pathogenesis of sepsis is largely attributed to inflammation, we hypothesized that lnc‐THRIL was correlated with inflammation markers levels in sepsis patients.^11^ In this study, we disclosed that lnc‐THRIL was positively correlated with CRP, PCT, TNF‐α, and IL‐1β levels in sepsis patients, which supported our hypothesis. The possible reasons were as follows: (a) Lnc‐THRIL could form a complex with hnRNPL and enhance the transcription of TNF‐α by binding to the promoter region of TNF‐α gene, which induced the increased release of TNF‐α‐related inflammatory cytokine IL‐1β and thereby enhanced the inflammation level of other inflammatory markers in sepsis patients. (b) Lnc‐THRIL was also reported to sponge several microRNAs such as miR‐99a, whose overexpression inhibited TNF‐α, IL‐6, IL‐1β, and MCP‐1 production.^10^, ^11^ Therefore, lnc‐THRIL might enhance inflammation levels by sponging anti‐inflammatory microRNAs in sepsis as well. However, this needed to be verified by further studies.

Additionally, the association of lnc‐THRIL with mortality in sepsis patients was assessed, which exhibited that lnc‐THRIL was upregulated in non‐survivors compared with survivors and was of good value in predicting mortality. The following subgroup analysis further validated the predictive value of lnc‐THRIL on mortality in sepsis patients. This might be due to that lnc‐THRIL facilitated inflammatory response by facilitating TNF‐α and other inflammatory markers, hence, aggravated the disease severity and increased the risk of ARDS, which eventually led to poor survival in sepsis patients. It was also interesting to note that lnc‐THRIL increased the mortality in both ARDS patients and non‐ARDS patients. Therefore, we speculated that lnc‐THRIL might increase the mortality in sepsis patients not only via raising the risk of ARDS, but also through other means, which needed to be further investigated.

This study remained several limitations. Firstly, this was a single center‐based study with small sample size, which might lead to selection bias and poor statistical power. Secondly, the mechanism of lnc‐THRIL in regulating inflammation was not investigated. Thirdly, sepsis patients were followed up in the hospital or until discharge in this study, which was a relatively short follow‐up period, and the survival of discharged patients needed to be evaluated as well.

In conclusion, lnc‐THRIL predicts increased risk of ARDS and positively correlates with disease severity, inflammation, and mortality in sepsis patients.

## CONFLICTS OF INTEREST

The authors declared no potential conflicts of interest with respect to the research, authorship, and/or publication of this article.
